# Overexpression of PPT2 Represses the Clear Cell Renal Cell Carcinoma Progression by Reducing Epithelial-to-mesenchymal Transition

**DOI:** 10.7150/jca.36477

**Published:** 2020-01-01

**Authors:** ChangFei Yuan, ZhiYong Xiong, Jian Shi, JingTao Peng, XianGui Meng, Cheng Wang, WenJun Hu, ZeYuan Ru, KaiRu Xie, HongMei Yang, Ke Chen, XiaoPing Zhang

**Affiliations:** 1 Department of Urology, Union Hospital, Tongji Medical College, Huazhong University of Science and Technology, Wuhan 430022, China.; 2 Department of Pathogenic Biology, School of Basic Medicine, Huazhong University of Science and Technology, Wuhan 430030, China.

**Keywords:** PPT2, ccRCC, EMT, Biomarker, Prognosis

## Abstract

Clear cell renal cell carcinoma (ccRCC) is one of the most common malignant tumors of the urinary system and has a poor response to radiotherapy and chemotherapy. To date, it is urgent to find effective biomarkers for the prevention and treatment of ccRCC. The occurrence and development of ccRCC is closely related to metabolic disturbances. Palmitoyl protein thioesterase 2 (PPT2) is a lysosomal thioesterase which is highly associated with metabolism, and it has never been studied in ccRCC. In this study, we first revealed PPT2 is significantly downregulated in ccRCC, and its expression level is highly correlated with clinicopathological parameters of ccRCC patients. Our ROC curve analyses evaluated the potential of PPT2 as a novel diagnostic marker and prognostic factor. Functional experiment results showed overexpression of PPT2 represses the proliferation, migration and invasion of ccRCC cells *in vitro*. Mechanistic investigations demonstrated that overexpression of PPT2 represses the ccRCC progression by reducing epithelial-to-mesenchymal transition (EMT). In conclusion, PPT2 is downregulated in ccRCC. Decreased PPT2 expression may be considered as a novel diagnostic marker and prognostic factor and serve as a therapeutic target for ccRCC.

## Introduction

Renal cell carcinomas (RCC) refers to approximately 90% of kidney cancers which arise from the renal parenchyma, and it accounts for 3% of all malignant tumors and 80%-85% of primary renal neoplasms respectively 1. The most common subtype of RCC is ccRCC which accounts for approximately 70%-80% of all RCC histological subtypes 2. Clinically, ccRCC frequently occurs with few symptoms or laboratory abnormalities, about one third of patients present with localized progression or distant metastasis at the time of diagnosis 3, 4. Patients with ccRCC usually are treated with standard surgical resections, but their outcomes are various. About 30% of ccRCC patients experience tumor recurrence or metastasis after surgical treatment, which remarkably reduces the likelihood of patients' survival 5. ccRCC is characterized by high metastasis risk, high rate of mortality, and poor response to radiotherapy and chemotherapy. Many advances have been made in the diagnosis and treatment of ccRCC in the recent decades. For example, targeted therapies have benefited lots of ccRCC patients due to the use of sunitinib and sorafenib. However, the majority of treated patients eventually suffer from tumor progression as a results of acquired resistance, and the incidence of ccRCC continues to increase 6, 7. Therefore, it is urgent to find effective biomarkers and prognostic indicators for prevention and evaluation of ccRCC, and a better understanding of the molecular mechanism underlying the occurrence and progression of ccRCC may contribute to the development of novel strategies for ccRCC treatment.

The main function of palmitoyl-protein thioesterase (PPT) is to cut off thioester linkage between a fatty acid and cysteine in lipid-modified proteins and remove long-chain fatty acids from cysteine residues in proteins 8, 9. It is reported that PPT is implicated in metabolism. PPT includes two types, PPT1 and PPT2, both of which play significant role in lysosomal thioester catabolism, and PPT1 shares 26% of identity in amino acid sequence with PPT2 10-12. PPT1 hydrolyzes thioester bonds that link fatty acids to cysteine residues in S-fatty acylated proteins 13, 14. It is a homolog of PPT2 and deficient in the lysosomal storage disorder, infantile neuronal ceroid lipofuscinosis (NCL) 15. PPT2 targets lysosomes through the mannose 6-phosphate receptor pathway just like PPT1 and is highly active against palmitoylated model substrates such as palmitoyl CoA 13, 16. Although they are very similar, PPT2 cannot rescue the neural inclusion phenotypes associated with loss of PPT1, which suggests distinct functions and substrates for these two thioesterases 17. At present, some researchers explored the possibility of regulating Ras tumorigenesis by targeting palmitoylation to disrupt the membrane interaction of specific Ras isoform 18. Studies have shown that, PPT1 promotes tumor progression and serves as the molecular target of drugs in cancer, targeting PPT1 blocks mTOR signaling and concurrently inhibits autophagy in a different way from catalytic inhibitors, thus provides a new strategy for cancer treatment 19, 20. However, the study of PPT2 in cancer has rarely been reported.

EMT is biological processes by which polarized epithelial cells interact by means of its basal surface with the basement membrane are transformed into mesenchymal phenotypic cells by specific procedures 21. This process plays an important role in tumorigenesis and cancer progression, wound healing and fibrosis, drug resistance and many other biological processes 22, 23. Additionally, EMT is closely related to increased cell migration and invasion capacity, cancer metastasis and resistance to apoptosis 24-26.

By analyzing The Cancer Genome Atlas (TCGA), we found that PPT2 mRNA expression is lower in ccRCC than in adjacent normal tissues, and the lower mRNA expression in ccRCC is closely related to the escalation of patients' clinicopathological parameters. Additionally, the expression of PPT2 can be used in the auxiliary diagnosis and prognosis prediction of ccRCC patients. Functional experiment results indicated that overexpression of PPT2 significantly represses the proliferation, migration and invasion of ccRCC cells by reducing EMT *in vitro*. In all, this study reveals a new diagnostic marker and prognostic factor for ccRCC, and it may provide new ideas and targets for the treatment of ccRCC.

## Materials and Methods

### Clinical renal cancer samples

Clinical renal cancer samples were acquired from patients who received partial or radical nephrectomy in the Department of Urology, Union Hospital, Tongji Medical College, Wuhan, China. 32 ccRCC samples were collected, and they all were paired with adjacent normal renal tissues. A part of the paired tissues were frozen in liquid nitrogen or -80℃ refrigerator for RNA extractions or Western Blot, the remaining tissues were fixed in 10% formalin for 24 hours at room temperature and then embedded in paraffin for IHC. None of those patients received preoperative adjuvant anticancer therapy. This study and experimental procedures were approved by the Human Research Ethics Committee of Huazhong University of Science and Technology (Wuhan, China), and written informed consent was obtained from all the patients involved.

### Cell culture

786-O, A498, ACHN, CAKi-1, OS-RC and 293 cell lines were gained from the American Type Culture Collection (Manassas, VA, USA), and all cells were cultured in Dulbecco's Modified Eagle Medium (DMEM) supplemented with 1% penicillin-streptomycin and 10% fetal bovine serum (FBS) with 5% CO_2_ at 37℃.

### Immunohistochemical staining assays

The collected ccRCC tissues and paired adjacent normal tissues were fixed in formalin and then embedded in paraffin. The paraffin-embedded cancer tissues were sectioned to a thickness of 4 μm. Subsequently, after deparaffinization, rehydration, blocking with hydrogen peroxide and antigen retrieval, these sections were incubated with primary rabbit anti-PPT2 polyclonal antibodies (A12251, ABclonal; Wuhan, China) overnight at 4℃. After washing three times with PBS, they were incubated with HRP-conjugated anti-rabbit secondary antibodies for 2 hours at room temperature. DAB was used for coloration, and dark brown was regarded as positive.

### Transient transfection assays

Plasmids overexpressing PPT2 were constructed in Vigene Biosciences (Shangdong, China) and then purchased. Cell lines A498 and CAKi-1 were seeded in 6-well plates at 50-70% confluence when being transfected. 3μg PPT2-overexpressing plasmid per well was added to the cells using Lipofectamine^TM^ 2000 reagents (Thermo Fisher Scientific; Waltham, USA). 24-48 hours after transfection, the cells were collected for efficiency determination and subsequent assays. All steps were carried out according to the manufacturer's instruction protocol.

### RNA extraction and qRT-PCR

Total RNA of ccRCC tissues and cell lines was extracted with the TRizol reagent (Thermo; Massachusetts, USA) according to the manufacturer's protocol. 1μg enriched tissue or cell RNA was used to synthesize cDNA through reverse transcription, which was accomplished by the RevertAid First-Strand cDNA Synthesis Kit (Thermo; Massachustts, USA). The Hieff^TM^qPCR SYBR**^R^**Green Master Mix (Thermo; Massachusetts, USA) was used for qPCR analysis by ABI ViiA7 qPCR System (Applied Biosystems; Foster, CA). Gene primers was purchased from RiboBio (Guangzhou, China), they were listed as follows: PPT2, left primer 5'-ACCATCCCAATGCCACAGTA-3'; right primer 5'-CAACCCAAAAGAATCCCGCA-3'. GAPDH, left primer 5'-CCAGAACAGCATCCCTGCCT-3'; right primer 5'-CCTGCTTCACCACCTTCTTG-3'.

### Western blot analysis

All the proteins of the processed tissues or cells were extracted with RIPA protein lysis buffer (Beyotime Institute of Biotechnology; Haimen, China) containing proportionately added protease inhibitor cocktail and phenylmethylsulfonyl fluoride (PMSF), then the impurities were removed after centrifugation at 15,000 rpm at 4°C. Subsequently, protein concentrations of the solution were measured by the Pierce^TM^ BCA Protein Assay Kit (Thermo; Massachusetts, USA). The calculated 30μg proteins per hole were separated in the 10% SDS-PAGE gel and transferred to the polyvinylidene fluoride (PVDF) membranes at 90 V for 90min. the PVDF membranes were washed softly for 5 min, and then blocked in PBST containing 5% non-fat milk for 2 hours at room temperature. The blocked membranes were washed, and cut into different strips according to the molecular weight of target proteins, then incubated with primary antibodies against target proteins overnight at 4℃. The next day, the membranes were incubated with secondary antibodies (1:2500, Invitrogen) for 2 hours at room temperature. Finally, the membranes were washed and used for detection. The primary antibodies used included anti-PPT2, anti-E-cadherin, anti-Vimentin, anti-β-actin and anti-Snail1.

### Cell proliferation assays

48 hours after transfection, A498 and CAKi-1 cells were added to 5 96-well plates at a density of 2,000 per well, the cell viability was detected after 0, 24, 48, 72 and 96 hours. Cell counting kit-8 (CCK-8) was used for cell proliferation rate determination according to the manufacturer's protocol. Specifically, 10 μl CCK-8 solution and 100 μl aforementioned media for cell culture were mixed and added to each well, the optical density of each well was measured at 450 nm after 4 hours. Each experiment was repeated three times independently.

### Migration and invasion assays

48 hours after transfection, the cells were collected and resuspended with medium without serum. 4×10^4^ cells in serum-free medium were seeded into the Boyden Transwell chambers (Corning; New York, USA) with 8-μg membrane filters for migration, while 8×10^4^ cells in serum-free medium were seeded into the aforementioned chambers which were pre-coated with Matrigel (Thermo Fisher Scientific; Waltham, USA) for invasion. The chambers were put into 24-well plates filled with complete medium with 10% FBS in advance. After 24 hours of incubation at 37℃ with 5% CO_2_, the cells on the upper surface were erased, while those invading on the lower surface were fixed in 100% methanol and stained with 0.05% crystal violet. Cells were counted in ten randomly chosen visual fields under a microscope. Each experiment was repeated three times independently.

### Statistical analysis

GraphPad Prism 5.0 and SPSS 18.0 software were used for all statistical analysis. The Student's t-test or Tukey's multiple comparisons test was used to assess differences of independent groups of continuous variables. Survival information was evaluated via Kaplan-Meier analysis and the log-rank test was used to assess the differences. χ2 tests were used to assess the significant correlation between PPT2 expression and clinicopathological parameters of ccRCC patients. ROC curve analysis was used to evaluate the diagnostic value of PPT2. A Cox proportional hazard regression model was used for univariate and multivariate analysis. Data are presented as mean ± SEM, p < 0.05 was considered statistically significant. P < 0.05, *; p < 0.01, **; p < 0.001, ***; p <0.0001, ****.

## Results

### PPT2 is downregulated in ccRCC and correlated with survival time of ccRCC patients

**Figure 1:**
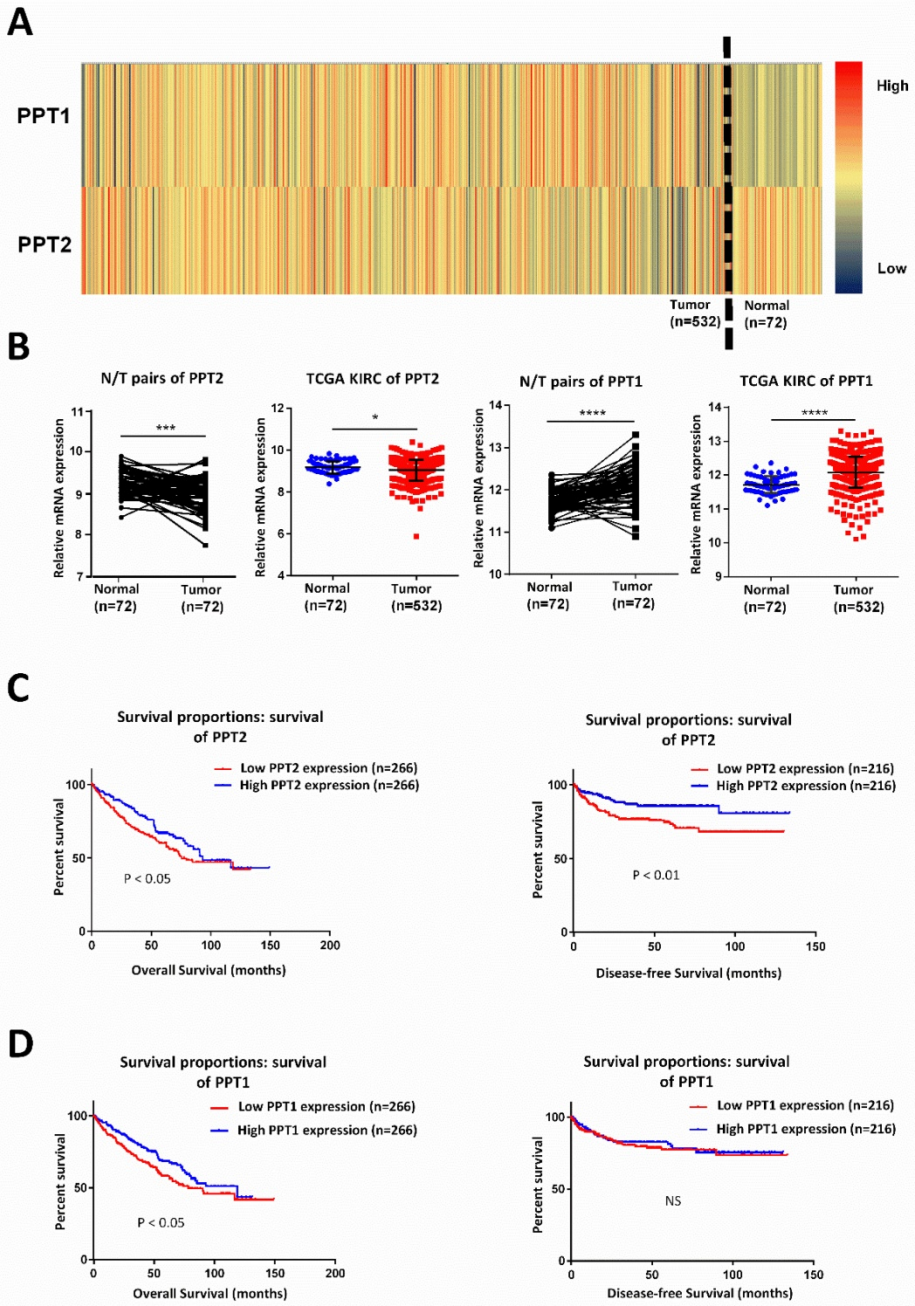
** PPT2 is downregulated in ccRCC and has significant correlations with patients' survival.** (A) Heat map depicting PPT family mRNA expression of all cases from TCGA database; red signifies high expression, yellow signifies medium expression, blue signifies low expression. (B) Comparison of PPT family mRNA expression in paired and in non-paired groups. (C-D) The mRNA expression of PPT2 and PPT1 was divided into low expression group and high expression group respectively on the basis of the median, the survival rate of two corresponding groups was evaluated with Kaplan Meier method and tested by using log rank test.

Lots of discoveries reveal that ccRCC can be regarded as a metabolism disease accompanied by reprogramming of carbohydrate and lipid and of the tricarboxylic acid cycle 27, 28. It is characterized by lipid accumulation in cancer cells, which indicating that metabolic disturbances are defining features of this tumor 29. PPT, which plays indispensable role in lysosomal thioester catabolism, is closely related to cell metabolism. Some researchers explored the possibility of regulating Ras tumorigenesis by targeting palmitoylation to disrupt the membrane interaction of specific Ras isoform 18. PPT1 promotes tumor progression and serves as the molecular target of drugs in cancer, targeting PPT1 blocks mTOR signaling and concurrently inhibits autophagy in a different way from catalytic inhibitors 19, 20. Therefore, we speculated that PPT family may play a potential role in ccRCC. By analyzing KIRC-TCGA database composed of 533 ccRCC cases, including 72 paired cases, we found that PPT family show differential expression in ccRCC. As showed in Figure 1A-B, PPT2 is downregulated in ccRCC while PPT1 showed an opposite result. Kaplan-Meier curves were used to analyze the correlation between gene expression and survival of patients. The results revealed that a lower PPT2 mRNA expression predicted a shorter survival time while the relationship between the expression of PPT1 and the survival time is not logically correct (Figure 1C-D). Additionally, we also analyzed the Overall Survival (OS) and Disease-free Survival (DFS) of both PPT1 and PPT2 in Gene Expression Profiling Interactive Analysis (GEPIA), and the analysis showed the same results from the TCGA KIRC datasets (Figure S1A-B). Therefore, PPT2 was chosen for subsequent study. According to the above results, we summarized that PPT2 is downregulated in ccRCC and correlated with survival time of ccRCC patients.

### Low PPT2 mRNA level is associated with various clinicopathological parameters in ccRCC patients

**Figure 2:**
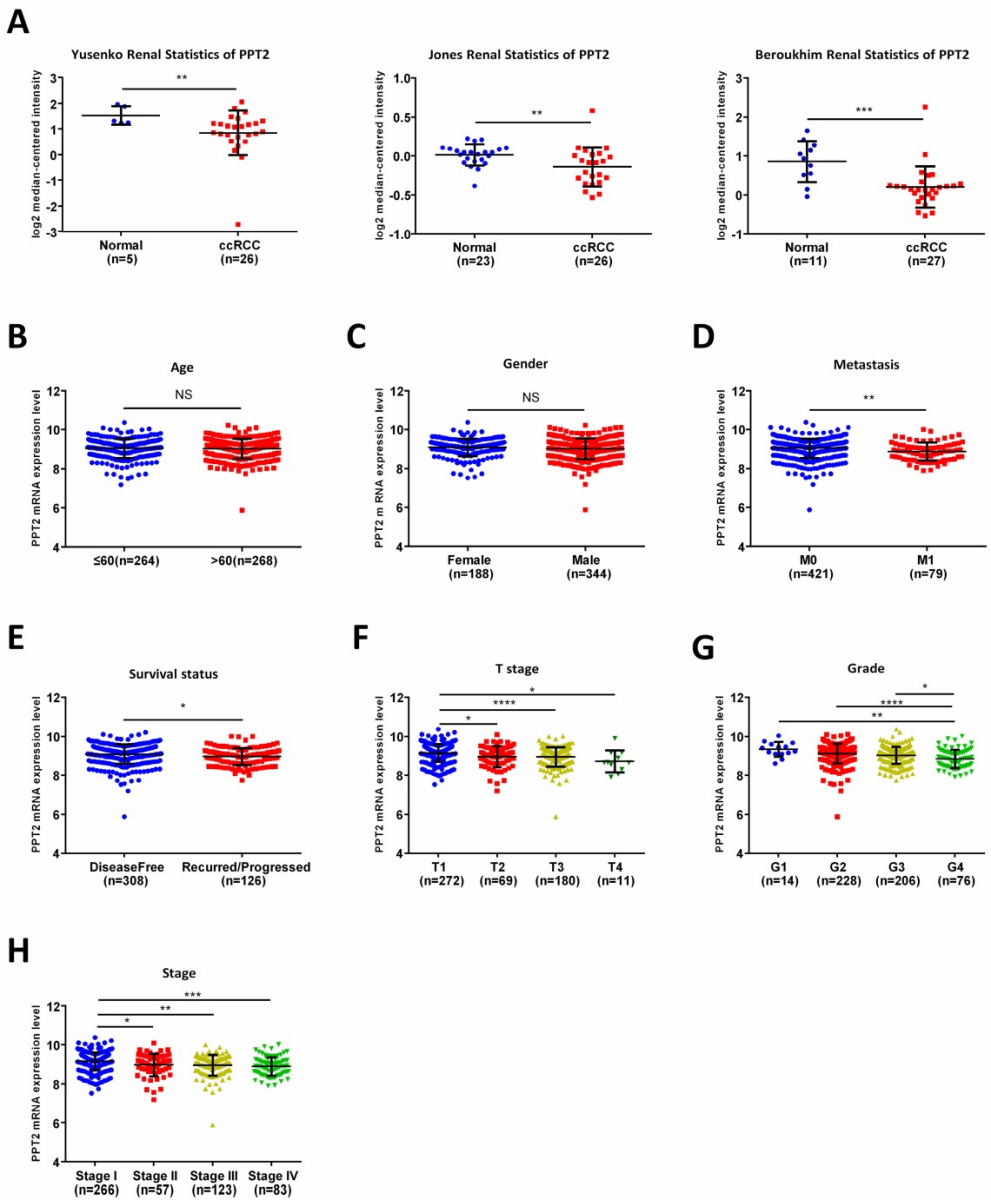
** PPT2 mRNA expression is lower in ccRCC comparing with normal tissues and is closely related to various clinicopathological parameters.** (A) The mRNA expression of PPT2 in statistics by Yusenko et al., Jones et al., and Beroukhim et al. were downloaded from Oncomine datasets and analyzed. In addition, the mRNA expression of PPT2 in ccRCC downloaded from the TCGA-KIRC datasets was compared with different clinicopathological parameters: (B) age, (C) gender, (D) metastasis, (E) survival status, (F) T stage, (G) pathological grade, (H) TNM stage, data differences were tested with Student's T-test.

To further analyze the relationship between PPT2 and ccRCC, Oncomine database was applied for study. As shown in Figure 2A, downregulation of PPT2 in ccRCC was confirmed by three additional data-sets over again. To figure out the correlations between mRNA expression of PPT2 and clinicopathological parameters of ccRCC patients, the TCGA database has been analyzed more deeply. The results revealed that the mRNA expression of PPT2 has no significant correlations with patients' age and gender (Figure 2B-C), while lower PPT2 mRNA expression was associated with worse M stage, survival status, T stage, pathological grades and TNM stage in ccRCC patients (Figure 2D-H). Additionally, a chi-square test based on TCGA database also suggested similar results (Table 1). Univariate and multivariate analysis were used to show the status of PPT2 expression in ccRCC risk factors. The results showed that PPT2 is a potential independent prognostic factor for ccRCC (Table 2). Therefore, we concluded that Low PPT2 mRNA level is associated with various clinicopathological parameters in ccRCC patients.

### Diagnostic value of PPT2 mRNA expression in ccRCC patients

**Figure 3:**
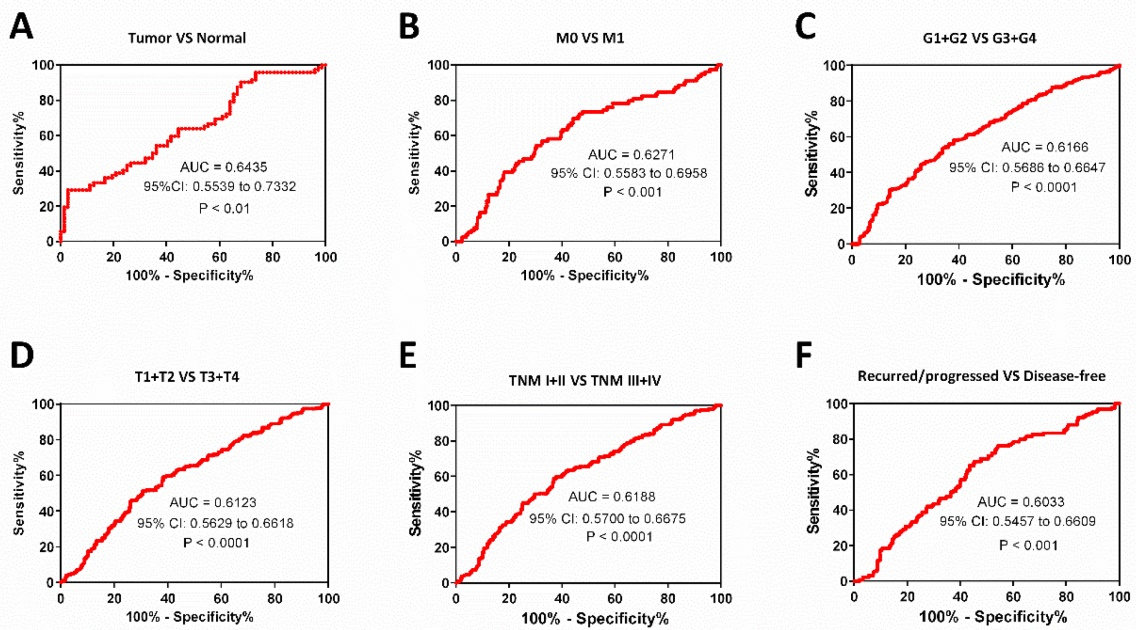
** The PPT2 expression may be considered as a diagnostic biomarker for ccRCC patients.** The ROC curve analyses of PPT2 mRNA expression in subgroups of ccRCC patients show PPT2 expression is of significance in distinguishing ccRCC from normal renal tissues (A), M0 from M1 (B), G1+G2 from G3+G4 (C), T1+T2 from T3+T4 (D), TNM I+II from TNM III+IV (E), the recurred/progressed from the disease-free (F).

As known, M stage, survival status, T stage, pathological grades and TNM stage are important clinical indicators to guide medication and surgical methods. We find low PPT2 mRNA level is associated with various clinicopathological parameters in ccRCC patients above, ROC curve analyses were used to evaluate diagnostic value of PPT2 mRNA expression level if it is regarded as a diagnostic marker and tested clinically. The results showed that PPT2 mRNA expression is of significance in differentiating ccRCC from normal tissues with an area under the curve (AUC) of 0.6435 (95% CI: 0.5539 to 0.7332, P<0.01) (Figure 3A), patients with metastasis from those without metastasis with an AUC of 0.6271 (95% CI: 0.5583 to 0.6958, P<0.001) (Figure 3B), G1+G2 from G3+G4 with an AUC of 0.6166 (95% CI: 0.5686 to 0.6647, P<0.0001) (Figure 3C), T1+T2 from T3+T4 with an AUC of 0.6123 (95% CI: 0.5629 to 0.6618, P<0.0001) (Figure 3D), TNM stage I+II from TNM stage III+IV with an AUC of 0.6188(95% CI: 0.5700 to 0.6675, P<0.0001) (Figure 3E), the recurred/progressed from the disease-free with an AUC of 0.6033 (95% CI: 0.5457 to 0.6609, P<0.001) (Figure 3F). In summary, PPT2 mRNA expression may be regarded as a diagnostic marker which is able to assist in identifying the progression of ccRCC.

### Downregulation of PPT2 is verified in ccRCC clinical samples and cell lines

**Figure 4:**
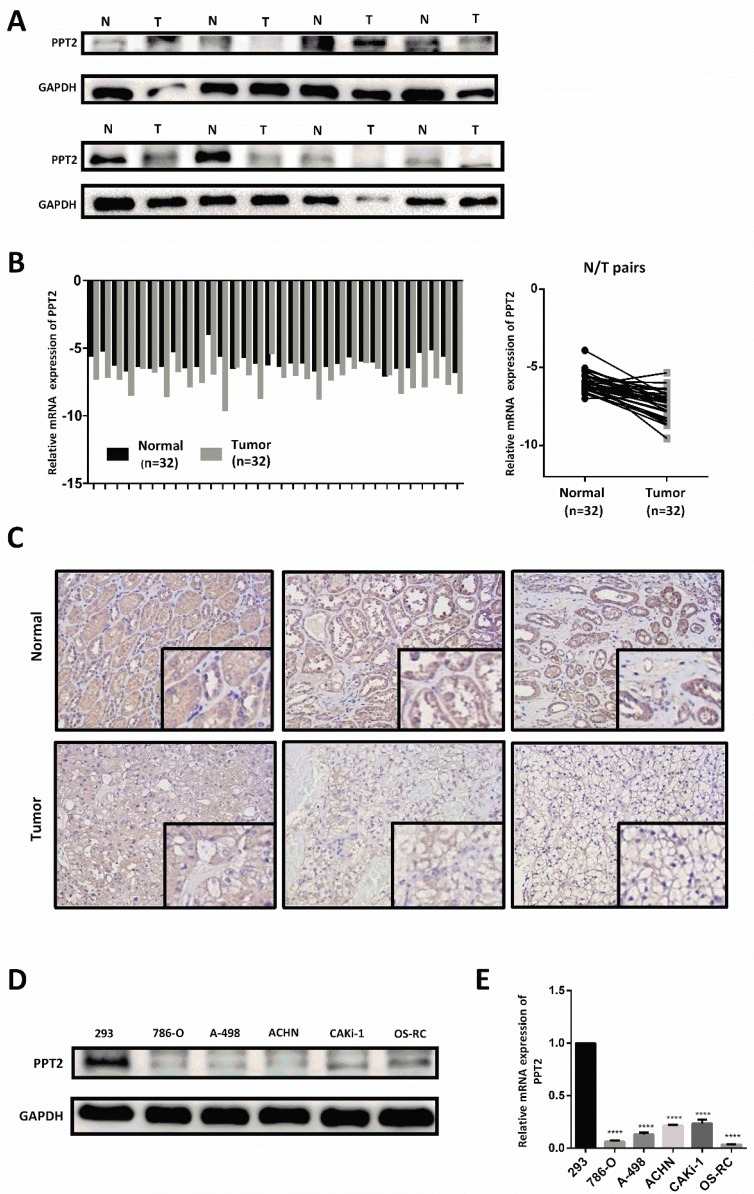
** PPT2 expression is downregulated both in clinical ccRCC samples and in renal cancer cell lines.** 32 pairs of postoperative clinical samples were collected, Western Blot (A), qRT-PCR (B) and IHC (C) were conducted in clinical ccRCC samples and in matched adjacent normal tissues. (D-E) Western Blot and qRT-PCR were also conducted to investigate PPT2 expression in cell lines 293, 786-O, A-498, ACHN, CAKi-1 and OS-RC.

To further confirm the results of bioinformatics analysis, our investigations were extended to assess mRNA and protein expression of PPT2 in ccRCC clinical samples and cell lines. As shown in Figure 4A-B, the results of Western Blot and Quantitative Real-time PCR (qRT-PCR) revealed that PPT2 is downregulated in ccRCC tissues collected from clinical application at both mRNA and protein levels (Figure 4A-B). Immunohistochemistry (IHC) was also performed to evaluate the expression of PPT2, similar results were acquired (Figure 4C). Furthermore, Western Blot and qRT-PCR were conducted in ccRCC cell lines, similarly, the results revealed that PPT2 is downregulated in ccRCC cell lines at both mRNA and protein levels (Figure 4D-E). As expected, PPT2 expression significantly decreased in ccRCC in the respect of cells and tissues.

### Overexpression of PPT2 represses proliferation, migration and invasion of ccRCC *in vitro*

**Figure 5:**
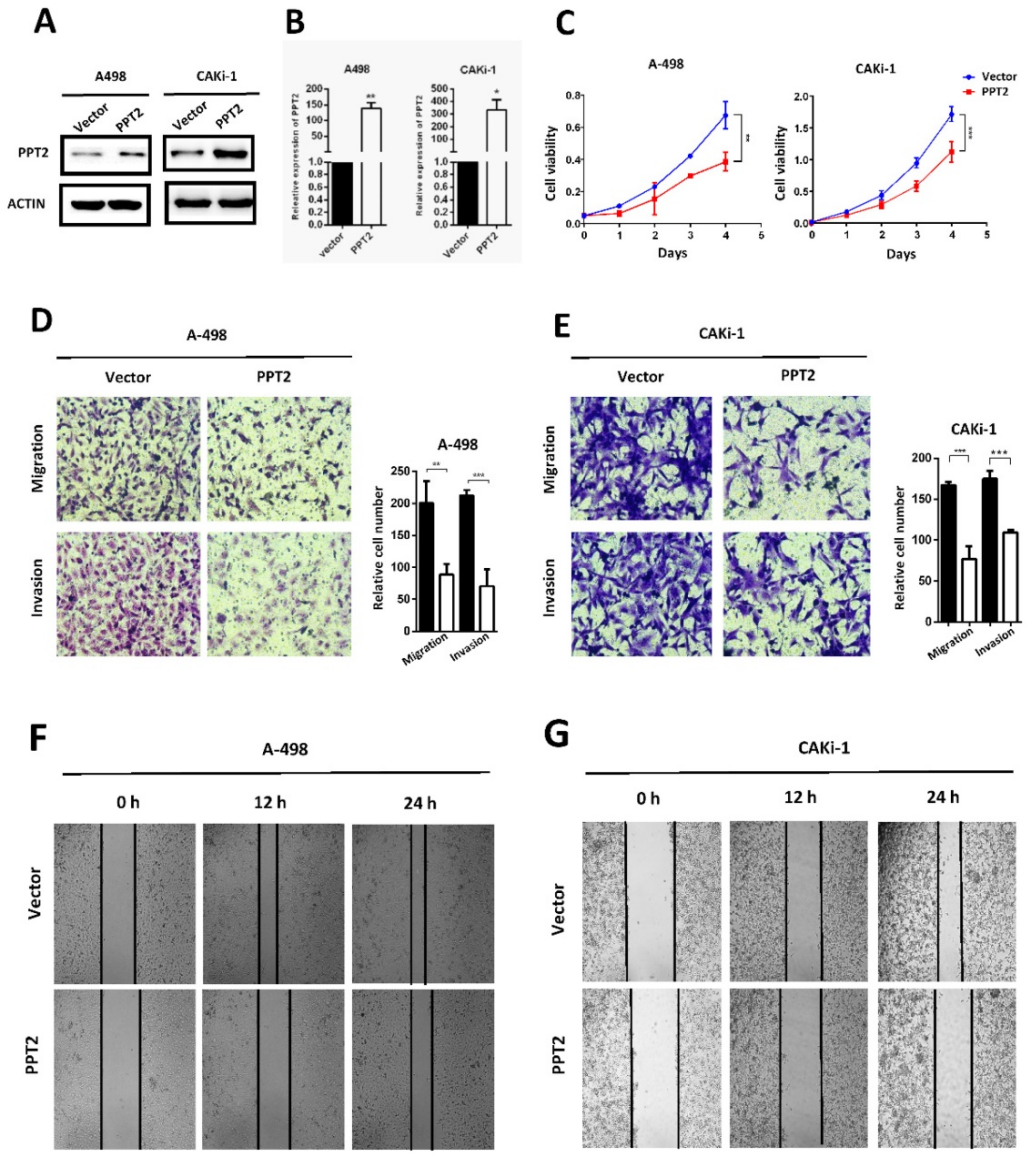
** Overexpression of PPT2 inhibits cell proliferation, migration and invasion *in vitro*.** (A-B) The efficiency of PPT2 overexpression in cell lines A-498 and CAKi-1 transfected with expression vectors of PPT2 was verified with Western Blot and qRT-PCR, relative gene expression was determined using the comparative delta-delta CT method. (C) CCK-8 assays revealed cell growth curves of A498 and CAKi-1 cells. (D-E) Migration and invasion assays for ccRCC cell lines A498 and CAKi-1, representative photographs were taken at ×200 magnification; number of cells was counted in ten random images from each group. (F-G) Representative micrographs of wound healing assays of cell lines A-498 and CAKi-1, wound closures were photographed at 0, 12 and 24 hours after wounding, representative photographs were taken at ×100 magnification.

The dysregulation of PPT2 in ccRCC may have a potential impact on the progression of ccRCC. To confirm this hypothesis, we successfully constructed A498 and CAKi-1 cell lines with overexpression of PPT2 by transfecting overexpression plasmid (Figure 5A-B). CCK-8 assays were conducted to evaluate the proliferation of cells. The results showed that overexpressing PPT2 significantly reduces the proliferation rate of ccRCC cells (Figure 5C). Migration and invasion are hallmarks of tumor progression as well. Transwell assays were performed to assess the migration and invasion ability of cells in this study. Our results revealed that migration and invasion of ccRCC cells were repressed following PPT2 overexpression (Figure 5D-E), and similar results were acquired in Wound healing assays (Figure 5F-G). In summary, overexpression of PPT2 represses proliferation, migration and invasion of ccRCC cells *in vitro*.

### Overexpression of PPT2 significantly reduces EMT in ccRCC cells

**Figure 6:**
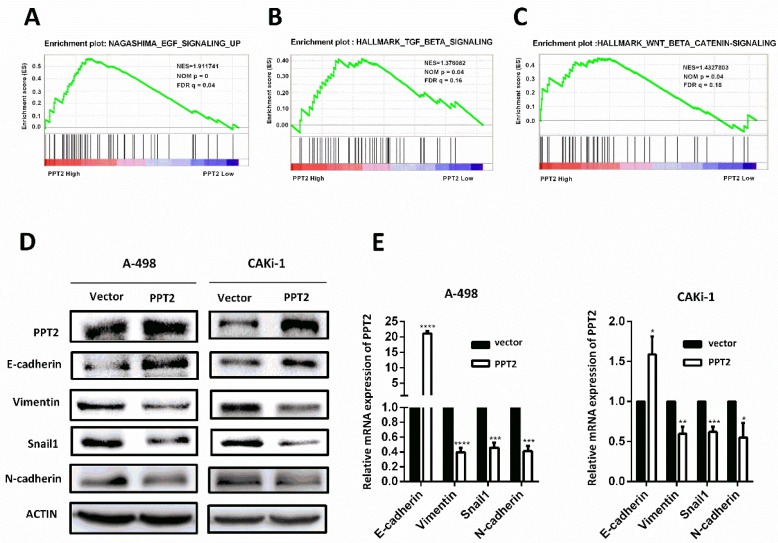
** Decreased expression of PPT2 facilitates EMT in ccRCC *in vitro*.** (A-C) GSEA was performed to investigate the biological pathways involved in PPT2 regulation on the basis of TCGA database. (D-E) The alterations of EMT markers (E-cadherin, Vimentin, Snail1 and N-cadherin) were tested with Western Blot and qRT-PCR.

To explore how PPT2 is involved in ccRCC tumorigenesis and progression, Gene Set Enrichment Analysis (GSEA) was performed to investigate the biological pathways involved on PPT2 regulation on the basis of TCGA database. The results revealed that PPT2 is highly associated with signatures of TGF-β signaling, WNT-β signaling and EGF signaling (Figure 6A-C). Many factors including epidermal growth factor (EGF), transforming growth factor-β (TGF-β), fibroblast growth factor (FGF), Wnt and Notch initiate the EMT process, and it is mediated via many EMT-related transcription factors such as Snail, Twist, Slug, and ZEB 30-32. EMT is an important factor in tumorigenesis and tumor progression and closely related to increased cell migration and invasion capacity, cancer metastasis and resistance to apoptosis 24-26. Thus, we speculated that PPT2 has an effect on EMT process. To test this hypothesis, the alterations of several EMT markers were evaluated in PPT2 overexpression cells. The results showed E-cadherin expression increased, while Vimentin, Snail1 and N-cadherin expression decreased in A498 and CAKi-1 cells transfected with PPT2 overexpression plasmids at both protein and mRNA levels (Figure 6D-E). In brief, overexpression of PPT2 inhibits EMT of ccRCC *in vitro*.

## Discussion

The occurrence and progression of tumors involve abnormalities in many signaling pathways. Currently, extensive gene expression and regulatory mechanisms in ccRCC remain unclear. In this study, through literature reading and database screening, we found a differentially expressed molecule in ccRCC, PPT2, which is closely correlated with cell metabolism. Further studies revealed PPT2 is downregulated in ccRCC, and low PPT2 expression is associated with various clinicopathological parameters of ccRCC patients. Functional experiment results indicated that overexpression of PPT2 remarkably represses the proliferation, migration and invasion of ccRCC *in vitro*. Mechanistic investigations demonstrated that PPT2 represses the ccRCC progression by reducing EMT. We finally came to the conclusion that PPT2 may be considered as a novel diagnostic marker and prognostic factor and provide a therapeutic target for ccRCC.

At present, many studies have revealed the dysfunction of metabolic pathways that controls biosynthesis and energetics in cancer. Metabolic disturbances and dysregulated metabolic pathways in cancers suggest that early diagnostic biomarkers can be identified and new therapeutic targets can be found for drug development which specially targeted to tumor metabolism rather than toxic to all proliferating cells. ccRCC can be regarded as a metabolism disease accompanied by reprogramming of carbohydrate and lipid and of the tricarboxylic acid cycle 27, 28. To date, the consensus seems to be that the mutation of von Hippel-Lindau (VHL) tumor suppressor gene and subsequent inactivation of hypoxia-inducible factor (HIF) lead to the occurrence of tumors 33. This process is implicated in changes of cell metabolism. HIF is affected by hypoxic environment. The activation of HIF signaling pathway changes the transcription of many metabolic enzymes, leads to metabolism reprogramming of cancer cells, especially the Warburg Effect in ccRCC 34, 35. Although a great number of studies about metabolic disturbance in ccRCC have been reported, the associations between metabolism and occurrence or progression of ccRCC are incompletely understood.

EMT is an important marker of tumorigenesis and progression and is closely related to increased cell migration and invasion capacity, cancer metastasis and resistance to apoptosis 24-26. On the basis of our experiment results linking EMT with PPT2 expression in ccRCC, we believe PPT2 plays an important role in the progression of ccRCC. Many factors initiate the EMT process, including epidermal growth factor (EGF), transforming growth factor-β (TGF-β), fibroblast growth factor (FGF), Wnt and Notch, and it is mediated via many EMT-related transcription factors such as Snail, Twist, Slug, and ZEB 30-32. Our study results revealed that PPT2 is highly associated with signatures of TGF-β signaling, WNT-β signaling and EGF signaling. TGF-β signal usually produces biological effects through the TGF-β/SMAD/Snail signaling pathway, for example, TGF-β induces the EMT process via this signaling pathway, leading to tumor occurrence and metastasis 36; EGF induces EMT via downregulation of E-cadherin caused by E-cadherin internalization and/or via upregulation of snail1 and/or TWIST, what's more, it can increase cell motility through extracellular matrix (ECM) degradation directed by MMP 37,38; for the canonical Wnt/β-catenin signaling pathway, lymphoid enhancer-binding factor 1 (LEF1) is a primary transcription factor that is involved in tumorigenesis and progression of multiple neoplasms 39, 40. Therefore, we hold that PPT2 is likely to affect EMT by regulating the above signaling pathways in ccRCC.

PPT is involved in cell metabolism by cutting off thioester linkage between a fatty acid and cysteine in lipid-modified proteins and removing long-chain fatty acids from cysteine residues in proteins 8, 9. PPT2 targets lysosomes through the mannose 6-phosphate receptor pathway and has strong activity against palmitoylated model substrates; it presumably plays a role in lysosomal thioester catabolism 13, 15, 16. According to previous results in this study, we guess that PPT2 plays a regulatory role in abnormal metabolism in ccRCC, especially lipid metabolism. The link between PPT2 and lipid metabolism in ccRCC is the focus of our next research.

In summary, PPT2 is downregulated in ccRCC, and decreased expression of PPT2 in ccRCC may promote tumor progression by facilitating EMT. Additionally, PPT2 may be a novel diagnostic marker and prognostic factor for ccRCC and provide new ideas for the treatment of ccRCC.

## Supplementary Material

Supplementary figure S1.

## Figures and Tables

**Table 1:** Clinicopathological parameters and their correlations with PPT2 mRNA expression

Parameter		PPT2 mRNA expression			P value
		Number	Low(n=266)	High(n=266)	
Age(years)	≤60	264	134	130	
	>60	268	132	136	.729
Gender	male	344	180	164	
	female	188	86	102	.147
Metastasis	M0	421	201	220	
	M1	79	58	21	.000*
	(MX)	(32)	(7)	(25)	
Survival status	Disease-free	308	134	174	
	Recurred/Progressed	126	80	46	.000*
	(Missing)	(98)	(52)	(46)	
T stage	T1+T2	343	147	196	
	T3+T4	189	119	70	.000*
Grade	G1+G2	242	96	146	
	G3+G4	282	165	117	.000*
	(GX)	(8)	(5)	(3)	
TNM stage	I+II	323	134	189	
	III+IV	209	132	77	.000*

**Table 2:** Univariate and multivariate analyses for patient overall survival

Risk factor	Univariate analysis			Multivariate analysis		
	HR	p-Value	95% CI	HR	p-Value	95% CI
PPT2 expression	0.712	0.030	0.525-0.967	0.942	0.047	0.687-1.292
Age	1.771	0.000	1.298-2.416	1.670	0.002	1.215-2.295
Gender	0.940	0.700	0.688-1.285	0.912	0.570	0.662-1.225
Metastasis	4.275	0.000	3.128-5.843	2.454	0.000	1.661-3.625
T stage	3.033	0.000	2.232-4.121	0.938	0.842	0.501-1.757
TNM stage	3.639	0.000	2.645-5.006	2.106	0.045	1.018-4.354
G stage	2.559	0.000	1.820-3.600	1.631	0.009	1.131-2.353

HR, hazard ratio, estimated from Cox proportional hazard regression model; CI, confidence interval of the estimated HR.

## Abbreviations

ccRCC: clear cell renal cell carcinoma
PPT2: palmitoyl protein thioesterase 2
EMT: epithelial-to-mesenchymal transition
RCC: renal cell carcinomas
NCL: neuronal ceroid lipofuscinosis
TCGA: The Cancer Genome Atlas
DMEM: Dulbecco's Modified Eagle Medium
FBS: fetal bovine serum
PMSF: phenylmethylsulfonyl fluoride
PVDF: polyvinylidene fluoride
qRT-PCR: Quantitative Real-time PCR
IHC: Immunohistochemistry
GSEA: Gene Set Enrichment Analysis
EGF: epidermal growth factor
TGF-β: transforming growth factor-β
FGF: fibroblast growth factor
ECM: extracellular matrix
LEF1: lymphoid enhancer-binding factor 1
