# Supplementary material for: Overexpression of PPT2 Represses the Clear Cell Renal Cell Carcinoma Progression by Reducing Epithelial-to-mesenchymal Transition
Source: J Cancer. 2020 Jan 1;11(5):1151–61. doi: 10.7150/jca.36477 (PMC6959065; doi:10.7150/jca.36477)

Supplementary material

Figure legend:

Figure S1. OS and DFS analysis of both PPT1 and PPT2 mRNA expression levels based on GEPIA. (A) OS and DFS analysis of PPT2 mRNA expression level based on GEPIA. (B) OS and DFS analysis of PPT1 mRNA expression level on the basis of GEPIA.

Supplementary Figure:

Figure S1

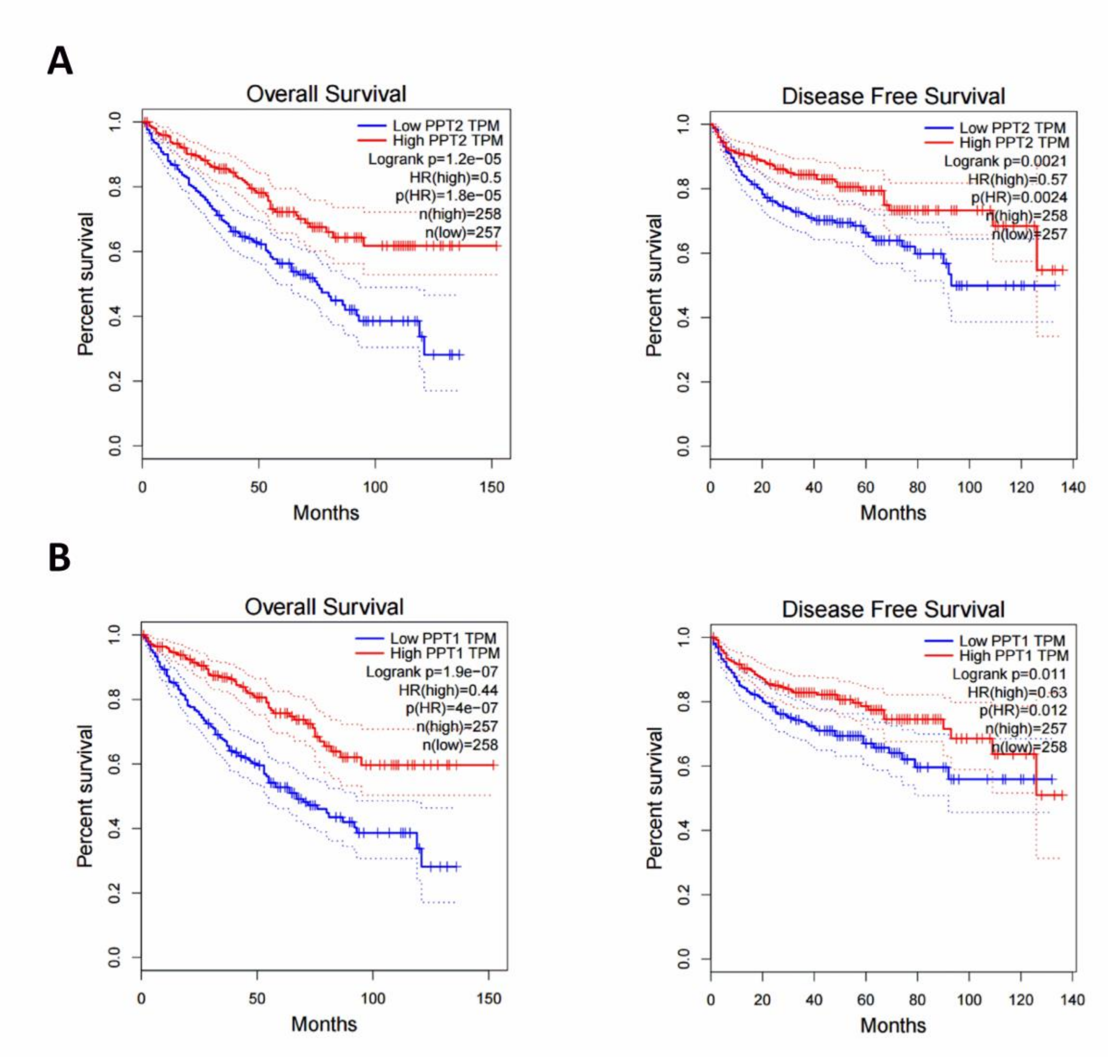

Supplement: Supplementary file 1 — Supplementary figure S1. [file jcav11p1151s1.pdf]
